# 2-Amino-5-methyl­pyridinium 4-carb­oxy­butano­ate

**DOI:** 10.1107/S1600536810024451

**Published:** 2010-06-26

**Authors:** Madhukar Hemamalini, Hoong-Kun Fun

**Affiliations:** a X-ray Crystallography Unit, School of Physics, Universiti Sains Malaysia, 11800 USM, Penang, Malaysia

## Abstract

In the title salt, C_6_H_9_N_2_^+^·C_5_H_7_O_4_^−^, the 2-amino-5-methyl­pyridinium cation is essentially planar, with a maximum deviation of 0.008 (1) Å. In the crystal, the protonated N atom and the 2-amino group are hydrogen bonded to the carboxyl­ate O atoms *via* a pair of N—H⋯O hydrogen bonds, forming an *R*_2_^2^(8) ring motif. The 4-carb­oxy­butano­ate anions are linked *via* O—H⋯O hydrogen bonds. The crystal structure is further stabilized by weak C—H⋯O inter­actions.

## Related literature

For background to the chemistry of substituted pyridines, see: Pozharski *et al.* (1997 ▶); Katritzky *et al.* (1996 ▶). For applications of glutaric acid, see: Windholz (1976 ▶); Saraswathi *et al.* (2001 ▶). For details of hydrogen bonding, see: Jeffrey & Saenger (1991 ▶); Jeffrey (1997 ▶); Scheiner (1997 ▶). For related structures, see: Hemamalini & Fun (2010**a* ▶,b* ▶); Fun *et al.* (2010 ▶). For hydrogen-bond motifs, see: Bernstein *et al.* (1995 ▶). For bond-length data, see: Allen *et al.* (1987 ▶). For the stability of the temperature controller used in the data collection, see: Cosier & Glazer (1986 ▶).
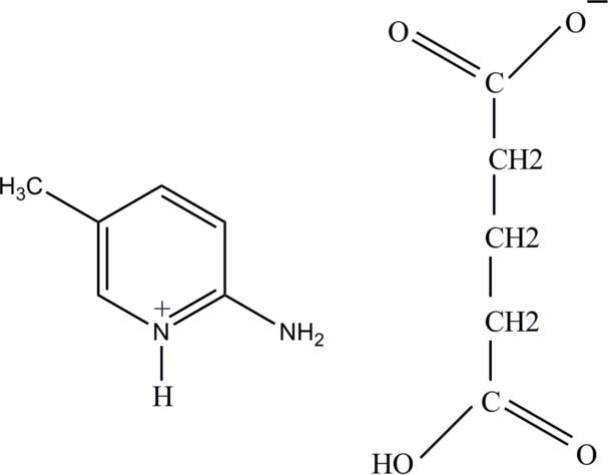


## Experimental

### 

#### Crystal data

C_6_H_9_N_2_^+^·C_5_H_7_O_4_^−^
*M* *_r_* = 240.26
Orthorhombic, 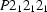

*a* = 5.3159 (10) Å
*b* = 14.383 (3) Å
*c* = 15.625 (3) Å
*V* = 1194.7 (4) Å^3^
*Z* = 4
Mo *K*α radiation
μ = 0.10 mm^−1^
*T* = 100 K
0.29 × 0.17 × 0.10 mm

#### Data collection

Bruker APEXII DUO CCD area-detector diffractometer
Absorption correction: multi-scan (*SADABS*; Bruker, 2009 ▶) *T*_min_ = 0.971, *T*_max_ = 0.990
7996 measured reflections
2028 independent reflections
1752 reflections with *I* > 2σ(*I*)
*R*_int_ = 0.037

#### Refinement

*R*[*F* ^2^ > 2σ(*F* ^2^)] = 0.037
*wR*(*F* ^2^) = 0.109
*S* = 1.05
2028 reflections
156 parameters
H-atom parameters constrained
Δρ_max_ = 0.23 e Å^−3^
Δρ_min_ = −0.20 e Å^−3^

### 

Data collection: *APEX2* (Bruker, 2009 ▶); cell refinement: *SAINT* (Bruker, 2009 ▶); data reduction: *SAINT*; program(s) used to solve structure: *SHELXTL* (Sheldrick, 2008 ▶); program(s) used to refine structure: *SHELXTL*; molecular graphics: *SHELXTL*; software used to prepare material for publication: *SHELXTL* and *PLATON* (Spek, 2009 ▶).

## Supplementary Material

Crystal structure: contains datablocks global, I. DOI: 10.1107/S1600536810024451/bv2141sup1.cif

Structure factors: contains datablocks I. DOI: 10.1107/S1600536810024451/bv2141Isup2.hkl

Additional supplementary materials:  crystallographic information; 3D view; checkCIF report

## Figures and Tables

**Table 1:** Hydrogen-bond geometry (Å, °)

*D*—H⋯*A*	*D*—H	H⋯*A*	*D*⋯*A*	*D*—H⋯*A*
N1—H1⋯O1^i^	0.86	1.82	2.672 (2)	170
N2—H2*A*⋯O2^i^	0.86	2.00	2.853 (3)	174
N2—H2*B*⋯O2	0.86	2.08	2.854 (2)	149
O4—H4⋯O1^ii^	0.82	1.76	2.5729 (19)	169
C2—H2⋯O3^iii^	0.93	2.59	3.441 (2)	152
C5—H5⋯O3^iv^	0.93	2.50	3.379 (2)	158

Symmetry codes: (i) 
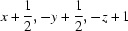
; (ii) 
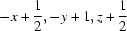
; (iii) 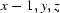; (iv) 
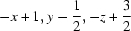
.

**Fig. 1.**
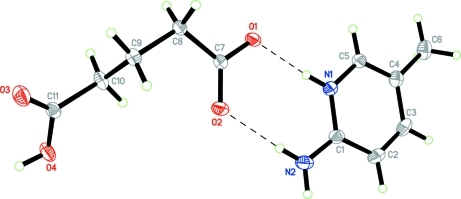
The asymmetric unit of the title compound. Displacement ellipsoids are drawn at the 50% probability level.

**Fig. 2.**
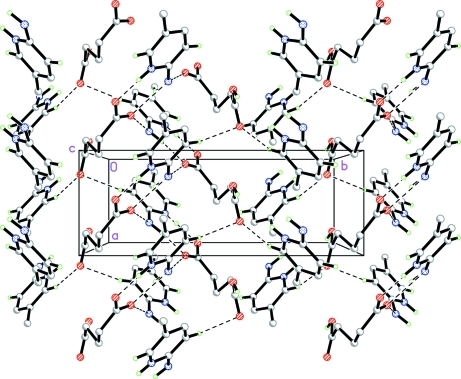
The crystal packing of the title compound, showing hydrogen-bonded (dashed lines) networks. H atoms are not involing the hydrogen bond interactions are omitted for clarity.

## supplementary crystallographic information

### Comment

Pyridine and its derivatives play an important role in heterocyclic chemistry (Pozharski *et al.*, 1997; Katritzky *et al.*, 1996). They are often involved in hydrogen-bond interactions (Jeffrey & Saenger, 1991; Jeffrey, 1997; Scheiner, 1997).  Glutaric acid (pentanedioic acid) is a dicarboxylic acid with five carbon atoms, occurring in plant and animal tissues. Glutaric acid is found in the blood and urine.  It is used in the synthesis of pharmaceuticals, surfactants and metal finishing compounds.  Alpha-ketoglutaric acid is used in dietary supplements to improve protein synthesis (Windholz, 1976). We have recently reported the crystal structures of 2-amino-5-methylpyridinium 4-nitrobenzoate (Hemamalini & Fun, 2010*a*), 2-amino-5-methylpyridinium nicotinate (Fun *et al.*, 2010) and 2-amino-5-methylpyridinium 3-aminobenzoate (Hemamalini & Fun, 2010*b*). In continuation of our studies of pyridinium derivatives, the crystal structure determination of the title compound has been undertaken.

The asymmetric unit (Fig. 1) contains a 2-amino-5-methylpyridinium cation and a 4-carboxybutanoate anion.  The 2-amino-5-methylpyridinium cation is essentially planar, with a maximum deviation of 0.008 (1) Å for atom N1. In the 2-amino-5-methylpyridinium cation, a wide angle (123.09 (16)°) is subtended at the protonated N1 atom.  The backbone conformation of the 4-carboxybutanoate anion can be described by the two torsion angles C11-C10-C9-C8 of -179.18 (14)° and C10-C9-C8-C7 of -72.74 (19)°. As evident from the torsion angles, the backbone is in a fully extended conformation (Saraswathi *et al.*, 2001) of the two carboxyl groups, one is deprotonated while the other is not.  The bond lengths (Allen *et al.*, 1987) and angles are within normal ranges.

In the crystal packing (Fig. 2), the protonated N1 atom and the 2-amino group (N2) is hydrogen-bonded to the carboxylate oxygen atoms (O1 and O2) via a pair of intermolecular N1—H1···O1 and N2—H2A···O2 hydrogen bonds forming a ring motif *R*_2_^2^(8) (Bernstein *et al.*, 1995). The 4-carboxybutanoate anions self-assemble via O4—H4···O1 hydrogen bonds. The crystal structure is further stabilized by weak C2—H2···O3 and C5—H5···O3 (Table 1) hydrogen bonds.

### Experimental

A hot methanol solution (20 ml) of 2-amino-5-methylpyridine (27 mg, Aldrich) and glutaric acid (33 mg, Merck) were mixed and warmed over a heating magnetic stirrer for a few minutes. The resulting solution was allowed to cool slowly at room temperature and crystals of the title compound appeared after a few days.

### Refinement

All hydrogen atoms were positioned geometrically [C–H = 0.93–0.97 Å, N–H = 0.86 Å and O–H = 0.82 Å] and were refined using a riding model, with U_iso_(H) = 1.2 U_eq_(C, N) or 1.5 U_eq_(O). The methyl H atoms were positioned geometrically and were refined using a riding model, with U_iso_(H) = 1.5U_eq_(C). A rotating group model was used for the methyl group. In the absence of significant anomalous scattering effects, 1457 Friedel pairs were merged.

### Crystal data

**Table:**

C_6_H_9_N_2_^+^·C_5_H_7_O_4_^−^	*F*(000) = 512
*M**_r_* = 240.26	*D*_x_ = 1.336 Mg m^−^^3^
Orthorhombic, *P*2_1_2_1_2_1_	Mo *K*α radiation, λ = 0.71073 Å
Hall symbol: P 2ac 2ab	Cell parameters from 2351 reflections
*a* = 5.3159 (10) Å	θ = 3.1–29.9°
*b* = 14.383 (3) Å	µ = 0.10 mm^−^^1^
*c* = 15.625 (3) Å	*T* = 100 K
*V* = 1194.7 (4) Å^3^	Block, colourless
*Z* = 4	0.29 × 0.17 × 0.10 mm

### Data collection

**Table:**

Bruker APEXII DUO CCD area-detector diffractometer	2028 independent reflections
Radiation source: fine-focus sealed tube	1752 reflections with *I* > 2σ(*I*)
graphite	*R*_int_ = 0.037
φ and ω scans	θ_max_ = 30.1°, θ_min_ = 2.8°
Absorption correction: multi-scan (*SADABS*; Bruker, 2009)	*h* = −7→7
*T*_min_ = 0.971, *T*_max_ = 0.990	*k* = −13→20
7996 measured reflections	*l* = −21→21

### Refinement

**Table:**

Refinement on *F*^2^	Primary atom site location: structure-invariant direct methods
Least-squares matrix: full	Secondary atom site location: difference Fourier map
*R*[*F*^2^ > 2σ(*F*^2^)] = 0.037	Hydrogen site location: inferred from neighbouring sites
*wR*(*F*^2^) = 0.109	H-atom parameters constrained
*S* = 1.05	*w* = 1/[σ^2^(*F*_o_^2^) + (0.0703*P*)^2^ + 0.0024*P*] where *P* = (*F*_o_^2^ + 2*F*_c_^2^)/3
2028 reflections	(Δ/σ)_max_ = 0.001
156 parameters	Δρ_max_ = 0.23 e Å^−^^3^
0 restraints	Δρ_min_ = −0.20 e Å^−^^3^

### Special details

**Table:**

Experimental. The crystal was placed in the cold stream of an Oxford Cryosystems Cobra open-flow nitrogen cryostat (Cosier & Glazer, 1986) operating at 100.0 (1) K.
Geometry. All s.u.'s (except the s.u. in the dihedral angle between two l.s. planes) are estimated using the full covariance matrix. The cell s.u.'s are taken into account individually in the estimation of s.u.'s in distances, angles and torsion angles; correlations between s.u.'s in cell parameters are only used when they are defined by crystal symmetry. An approximate (isotropic) treatment of cell s.u.'s is used for estimating s.u.'s involving l.s. planes.
Refinement. Refinement of F^2^ against ALL reflections. The weighted R-factor wR and goodness of fit S are based on F^2^, conventional R-factors R are based on F, with F set to zero for negative F^2^. The threshold expression of F^2^ > 2σ(F^2^) is used only for calculating R-factors(gt) etc. and is not relevant to the choice of reflections for refinement. R-factors based on F^2^ are statistically about twice as large as those based on F, and R- factors based on ALL data will be even larger.

### Fractional atomic coordinates and isotropic or equivalent isotropic displacement parameters (Å^2^)

**Table:**

	*x*	*y*	*z*	*U*_iso_*/*U*_eq_	
N1	0.1366 (3)	0.23633 (10)	0.72155 (9)	0.0178 (3)	
H1	0.2568	0.1970	0.7149	0.021*	
N2	0.2291 (4)	0.29299 (12)	0.58707 (9)	0.0238 (4)	
H2A	0.3451	0.2516	0.5828	0.029*	
H2B	0.2030	0.3313	0.5457	0.029*	
C1	0.0899 (4)	0.29783 (12)	0.65773 (10)	0.0187 (4)	
C2	−0.1037 (4)	0.36383 (13)	0.67157 (11)	0.0232 (4)	
H2	−0.1392	0.4084	0.6301	0.028*	
C3	−0.2378 (4)	0.36172 (14)	0.74603 (12)	0.0237 (4)	
H3	−0.3653	0.4051	0.7543	0.028*	
C4	−0.1888 (4)	0.29528 (12)	0.81139 (11)	0.0198 (4)	
C5	0.0019 (4)	0.23393 (12)	0.79573 (10)	0.0184 (3)	
H5	0.0408	0.1894	0.8368	0.022*	
C6	−0.3412 (4)	0.29045 (14)	0.89247 (13)	0.0266 (4)	
H6A	−0.5064	0.2673	0.8797	0.040*	
H6B	−0.3544	0.3514	0.9170	0.040*	
H6C	−0.2601	0.2495	0.9324	0.040*	
O1	−0.0252 (3)	0.40052 (9)	0.28998 (7)	0.0196 (3)	
O2	0.0882 (3)	0.35424 (10)	0.42007 (7)	0.0238 (3)	
O3	0.7031 (3)	0.57159 (10)	0.58440 (8)	0.0245 (3)	
O4	0.3325 (3)	0.54500 (10)	0.64750 (8)	0.0240 (3)	
H4	0.4108	0.5588	0.6909	0.036*	
C7	0.1204 (4)	0.40279 (12)	0.35520 (10)	0.0160 (3)	
C8	0.3451 (4)	0.46878 (12)	0.34924 (10)	0.0172 (3)	
H8A	0.4587	0.4460	0.3054	0.021*	
H8B	0.2852	0.5294	0.3311	0.021*	
C9	0.4914 (4)	0.48016 (12)	0.43228 (10)	0.0177 (3)	
H9A	0.6509	0.5102	0.4205	0.021*	
H9B	0.5261	0.4194	0.4565	0.021*	
C10	0.3435 (4)	0.53813 (13)	0.49684 (10)	0.0191 (4)	
H10A	0.3062	0.5982	0.4717	0.023*	
H10B	0.1847	0.5074	0.5086	0.023*	
C11	0.4815 (4)	0.55288 (11)	0.58018 (10)	0.0167 (3)	

### Atomic displacement parameters (Å^2^)

**Table:**

	*U*^11^	*U*^22^	*U*^33^	*U*^12^	*U*^13^	*U*^23^
N1	0.0184 (8)	0.0183 (7)	0.0169 (6)	0.0033 (6)	−0.0006 (6)	0.0006 (5)
N2	0.0275 (9)	0.0264 (8)	0.0176 (6)	0.0062 (7)	−0.0001 (7)	0.0053 (5)
C1	0.0203 (9)	0.0176 (7)	0.0181 (7)	−0.0002 (7)	−0.0050 (7)	0.0006 (6)
C2	0.0236 (9)	0.0193 (8)	0.0268 (8)	0.0038 (8)	−0.0040 (8)	0.0049 (7)
C3	0.0194 (9)	0.0179 (8)	0.0339 (9)	0.0052 (8)	−0.0008 (9)	−0.0002 (7)
C4	0.0185 (9)	0.0177 (7)	0.0233 (8)	−0.0028 (7)	0.0012 (8)	−0.0017 (6)
C5	0.0206 (9)	0.0175 (7)	0.0170 (7)	−0.0001 (7)	−0.0010 (7)	−0.0002 (5)
C6	0.0259 (10)	0.0230 (9)	0.0308 (9)	−0.0021 (9)	0.0070 (9)	−0.0045 (7)
O1	0.0215 (7)	0.0246 (6)	0.0126 (5)	−0.0056 (6)	−0.0017 (5)	0.0022 (4)
O2	0.0298 (8)	0.0269 (6)	0.0146 (5)	−0.0080 (6)	−0.0044 (6)	0.0058 (5)
O3	0.0200 (7)	0.0339 (7)	0.0195 (6)	−0.0022 (6)	−0.0026 (6)	−0.0033 (5)
O4	0.0230 (7)	0.0354 (7)	0.0136 (5)	−0.0054 (6)	−0.0003 (6)	−0.0058 (5)
C7	0.0175 (8)	0.0166 (7)	0.0138 (6)	−0.0002 (7)	0.0006 (7)	−0.0015 (5)
C8	0.0176 (8)	0.0204 (8)	0.0136 (6)	−0.0029 (7)	0.0003 (7)	−0.0017 (6)
C9	0.0172 (8)	0.0196 (7)	0.0163 (7)	0.0013 (7)	−0.0008 (7)	−0.0027 (6)
C10	0.0182 (9)	0.0236 (8)	0.0155 (7)	0.0037 (7)	−0.0045 (7)	−0.0054 (6)
C11	0.0198 (8)	0.0154 (7)	0.0150 (6)	0.0028 (7)	−0.0022 (7)	−0.0012 (5)

### Geometric parameters (Å, °)

**Table:**

N1—C1	1.356 (2)	C6—H6C	0.9600
N1—C5	1.363 (2)	O1—C7	1.280 (2)
N1—H1	0.8600	O2—C7	1.243 (2)
N2—C1	1.331 (2)	O3—C11	1.210 (2)
N2—H2A	0.8600	O4—C11	1.321 (2)
N2—H2B	0.8600	O4—H4	0.8200
C1—C2	1.417 (3)	C7—C8	1.528 (3)
C2—C3	1.365 (3)	C8—C9	1.522 (2)
C2—H2	0.9300	C8—H8A	0.9700
C3—C4	1.423 (3)	C8—H8B	0.9700
C3—H3	0.9300	C9—C10	1.527 (2)
C4—C5	1.366 (3)	C9—H9A	0.9700
C4—C6	1.505 (3)	C9—H9B	0.9700
C5—H5	0.9300	C10—C11	1.509 (2)
C6—H6A	0.9600	C10—H10A	0.9700
C6—H6B	0.9600	C10—H10B	0.9700
			
C1—N1—C5	123.09 (16)	H6B—C6—H6C	109.5
C1—N1—H1	118.5	C11—O4—H4	109.5
C5—N1—H1	118.5	O2—C7—O1	123.48 (17)
C1—N2—H2A	120.0	O2—C7—C8	120.42 (15)
C1—N2—H2B	120.0	O1—C7—C8	116.09 (14)
H2A—N2—H2B	120.0	C9—C8—C7	114.48 (13)
N2—C1—N1	118.31 (16)	C9—C8—H8A	108.6
N2—C1—C2	124.45 (16)	C7—C8—H8A	108.6
N1—C1—C2	117.24 (16)	C9—C8—H8B	108.6
C3—C2—C1	119.66 (16)	C7—C8—H8B	108.6
C3—C2—H2	120.2	H8A—C8—H8B	107.6
C1—C2—H2	120.2	C8—C9—C10	111.04 (15)
C2—C3—C4	122.08 (18)	C8—C9—H9A	109.4
C2—C3—H3	119.0	C10—C9—H9A	109.4
C4—C3—H3	119.0	C8—C9—H9B	109.4
C5—C4—C3	116.18 (16)	C10—C9—H9B	109.4
C5—C4—C6	121.36 (16)	H9A—C9—H9B	108.0
C3—C4—C6	122.44 (17)	C11—C10—C9	113.37 (15)
N1—C5—C4	121.73 (16)	C11—C10—H10A	108.9
N1—C5—H5	119.1	C9—C10—H10A	108.9
C4—C5—H5	119.1	C11—C10—H10B	108.9
C4—C6—H6A	109.5	C9—C10—H10B	108.9
C4—C6—H6B	109.5	H10A—C10—H10B	107.7
H6A—C6—H6B	109.5	O3—C11—O4	123.98 (16)
C4—C6—H6C	109.5	O3—C11—C10	123.45 (17)
H6A—C6—H6C	109.5	O4—C11—C10	112.56 (15)
			
C5—N1—C1—N2	−178.57 (16)	C3—C4—C5—N1	−0.2 (3)
C5—N1—C1—C2	2.0 (3)	C6—C4—C5—N1	178.30 (16)
N2—C1—C2—C3	178.99 (19)	O2—C7—C8—C9	−9.4 (2)
N1—C1—C2—C3	−1.7 (3)	O1—C7—C8—C9	171.50 (15)
C1—C2—C3—C4	0.4 (3)	C7—C8—C9—C10	−72.74 (19)
C2—C3—C4—C5	0.5 (3)	C8—C9—C10—C11	−179.18 (14)
C2—C3—C4—C6	−177.97 (18)	C9—C10—C11—O3	42.8 (2)
C1—N1—C5—C4	−1.1 (3)	C9—C10—C11—O4	−138.56 (16)

### Hydrogen-bond geometry (Å, °)

**Table:**

*D*—H···*A*	*D*—H	H···*A*	*D*···*A*	*D*—H···*A*
N1—H1···O1^i^	0.86	1.82	2.672 (2)	170
N2—H2A···O2^i^	0.86	2.00	2.853 (3)	174
N2—H2B···O2	0.86	2.08	2.854 (2)	149
O4—H4···O1^ii^	0.82	1.76	2.5729 (19)	169
C2—H2···O3^iii^	0.93	2.59	3.441 (2)	152
C5—H5···O3^iv^	0.93	2.50	3.379 (2)	158

Symmetry codes: (i) *x*+1/2, −*y*+1/2, −*z*+1; (ii) −*x*+1/2, −*y*+1, *z*+1/2; (iii) *x*−1, *y*, *z*; (iv) −*x*+1, *y*−1/2, −*z*+3/2.
